# Effects of different boiling and stir-fried with bran processes on chemical composition of *Pueraria lobata*

**DOI:** 10.3389/fnut.2025.1605521

**Published:** 2025-06-09

**Authors:** Mao-Cheng Tang, Xin-Ying Song, Wei Huang, Xiang-Yang Lu, Chong Wang, Hu-Hu Liu

**Affiliations:** College of Bioscience and Biotechnology, Hunan Agricultural University, Changsha, Hunan, China

**Keywords:** *Pueraria lobata*, boiling process, frying processes, heavy metals, food-medicine homology

## Abstract

**Background:**

*Pueraria lobata* has a long edible history owing to its special physiological activities. However, research on the processing methods for *P. lobata* is limited.

**Methods:**

In this study, a more suitable boiling process was established by optimizing the heating method, solvent selection, and different processing techniques. In addition, the stir-fried with bran method was used to investigate the changes in heavy metal contents in *P. lobata*.

**Results:**

Compared with the traditional process, the heavy metal content was reduced 96.5% and the isoflavone content was increased 7.5% in the decoction obtained by continuous heating with a ratio of water-to-white wine of 10:1. Meanwhile, we explored the effect of different stir-fried with bran processes on the reduction of lead, cadmium, chromium, copper, and arsenic in *P. lobata*. The results showed that the use of medium heat for 20 s and 30% bran roasting for 1 min resulted in the lowest overall heavy metal content in *P. lobata*.

**Conclusion:**

This study will provide the reference for reducing heavy metals from *P. lobata* using different processing methods.

## Graphical abstract

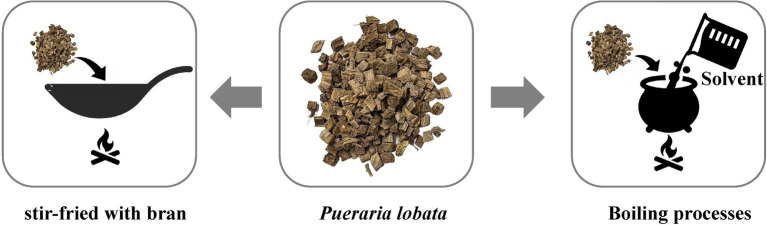

## 1 Introduction

*Pueraria lobata*, a medicinal and food homologous plant with a long edible history, is first recorded in “Shen Nong’s Herbal Classic” (1). *P. lobata* is produced in most areas of China, including Fujian, Guangdong, Guangxi, Jiangxi, Hunan, Hubei, Gansu, and other provinces (2). Previously, Song et al. showed that the chemical components of *P. lobata* include flavonoids, triterpenes, and other compounds (3). In 2000, *P. lobata* received official approval from the National Health Commission of China (4).

Heavy metals are considered a safety issue of traditional Chinese medicine (5). Atmospheric, water, and soil conditions play substantial roles in the cultivation and growth of medicinal herbs (6). For example, Zhang et al. investigated and analyzed the distribution of major metallic elements in *P. lobata* from Dali, revealing the presence of heavy metals, such as iron, chromium, zinc, copper, and lead in *P. lobata* (7). In general, the cultivation environment of medicinal and edible herbs is prone to contamination by various heavy metals. Previously, Chen et al. investigated five heavy metals (Pb, As, Hg, Cd, Cu) in 1773 medicinal herbs from the soil, and found that the metal content in 541 samples (30.51%) exceeded the standards set by the Chinese Pharmacopoeia (2020 edition) (8).

The common processing methods for foods and medicinal herbs include drying, stir-frying, boiling, and cutting. Mainly, the commonly used processing methods for *P. lobata* include drying, stir-frying (with wheat bran or honey bran), boiling (vinegar processing), and cutting (9). Chinese herbal medicines need specific processing methods to meet the quality standards for clinical use in traditional Chinese medicine (10). Zhong et al. demonstrated how various cutting specifications and processing methods affect the flavonoid content in *P. lobata* and *Pueraria* powders (11). In that research, it was indicated that after stewing or vinegar processing, the levels of 3′-hydroxy-puerarin, daidzin, and 3′-methoxy-puerarin in *P. lobata* slices decreased, while the levels of daidzein and puerarin increased (11). Recently, He et al. developed an optimized cooking process to prepare a more nutritious *Lilii Bulbus* soup, leading to a 54% increase in polysaccharide content and a 33.5% reduction in total heavy metal content (12). Therefore, it is necessary to reduce the heavy metal content while ensuring the retention of the nutrient content of *P. lobata*.

To reduce the heavy metal content in *P. lobata* while retaining the bioactive ingredients (polysaccharides and flavonoids), this study aimed to establish a more suitable boiling process by optimizing the heating method, solvent selection, and different processing techniques. In addition, the stir-fried with bran method was used to investigate the changes in heavy metal contents in *P. lobata*. This study aims to provide new insights into the processing methods of *P. lobata*.

## 2 Materials and methods

### 2.1 Preparation of materials

*Pueraria lobata* was purchased from QianCao Chinese Herbal Pieces Co. Ltd., Beijing, China. White wine (38% strength) was sourced from Shunxin Agriculture Co. Ltd., Beijing, China. White vinegar was purchased from Xianglian Sauce and Food Co. Ltd., Changsha, China. The total polysaccharide content was measured using a Total Polysaccharide Determination Kit (Keming Biotechnology Co., Ltd., Suzhou, China). Decoction pots were purchased from Joyoung Co., Ltd., Jinan, China. A digital electric heater was purchased from LiChen Instrument Technology Co., Ltd., Shanghai, China.

### 2.2 Boiling processes of *Pueraria lobata*

Traditional process: (1) Distilled water was used as the solvent for decoction. (2) 50 g of *P. lobata* slices were purified to remove impurities and soaked in distilled water for 30 min. (3) 50 g of *P. lobata* slices that had been soaked in Step (2) were placed in 500 mL of the decoction prepared in Step (1) and boiled for 50 min. The mixture was boiled at high heat in a decoction pot for 30 min, then simmering at low heat for an additional 20 min. The decoction was separated and stored. The residual herbal material from the initial decoction was reintroduced into the decoction obtained in step (2) and boiled for 40 min. The mixture was heated for 25 min, then stewed at low heat for 15 min in a decoction pot. The decoction was then strained. Finally, the two batches of decoction were combined to obtain a *P. lobata* decoction (13, 14).

Processing method 1 (PM1): (1) Distilled water and edible white wine were prepared in a volume ratio of 10:1 to form a decoction solution. Steps (2) and (3) were performed according to the traditional process.

Processing method 2 (PM2): (1) Distilled water and white vinegar were prepared at a volume ratio of 10:1; Steps (2) and (3) were performed according to the traditional process.

Processing method 3 (PM3): (1) Distilled water was used as the solvent for decoction. (2) 50 g of *P. lobata* slices were soaked in distilled water for 30 min. (3) After soaking as described in step (2), 50 g of *P. lobata* slices were boiled in the 500 mL decoction solution obtained from step (1) for 50 min. The medicinal liquid was removed to obtain a decoction from *P. lobata* slices. The boiling temperature was maintained at 100°C, and an electric heater was utilized for the boiling process.

Processing method 4 (PM4): (1) Distilled water and edible white wine were prepared according to the volume ratio of 10:1; Steps (2) and (3) mirror those outlined in treatment process 3.

Processing method 5 (PM5): (1) Distilled water and edible white vinegar were prepared at a volume ratio of 10:1. The procedures outlined in steps (2) and (3) were identical to those detailed in treatment Process 3.

### 2.3 Extraction and determination of polysaccharide content

The total content of polysaccharides in *P. lobata* slices was extracted according to a previously reported method (15). In this process, 1 mL of *P. lobata* decoction was used, and 1 mL of water was added to dissolve the polysaccharides. The extract was heated in a metal bath at 100°C for 2 h, then centrifuged at 10000 g for 10 min after cooling, and the supernatant was collected. 200 μL of supernatant was taken and mixed thoroughly with 800 μL of ethanol. After standing overnight at 4°C, 100 μL of Reagent 1 and 0.5 mL of concentrated sulfuric acid were added and incubated in a water bath at 90°C, then cooled with tap water. The absorbance (A) was measured at 490 nm, and the polysaccharide content was determined according to the manufacturer’s instructions (12).

### 2.4 Analysis of heavy metal content

A boiling tube containing 1 mL of decoction and 10 mL of acid mixture (GR grade HNO_3_: GR grade HClO_4_ = 4:1) was kept at 25°C for 16 h, then heated to 95°C and kept at this temperature in a water bath for 2 h until the digested sample was transparent. The cooling volume was fixed at 25 mL with pure water before filtration. The digested samples were analyzed using Inductively Coupled Plasma Mass Spectrometry. The reagent blanks were treated the same way as the samples (12).

### 2.5 Extraction and determination of isoflavones

For sample preparation, *P. lobata* was powdered using a high-speed tissue disintegrator and then homogenized with a standard sieve having a pore size of 0.83 mm. The *P. lobata* sample (1.0 g) was weighed in a beaker, and 50 mL of 70% ethanol solution was added. After 25 min of ultrasonic extraction, the mixture was filtered to collect the filtrate for further processing. The isoflavone content of *P. lobata* and its presence in the decoction were determined using an ultraviolet spectrophotometer (16). The puerarin stock solution was accurately pipetted and diluted with 70% ethanol to concentrations of 0.005, 0.01, 0.025, 0.05, and 0.1 mg/mL to serve as puerarin standard solutions. A 70% ethanol solution was used as a blank control group. The absorbance was measured at a wavelength of 250 nm.

### 2.6 Orthogonal design of *Pueraria lobata* stir-fried with bran process

The process of stir-fried begins with preheating the bran in decoction pot, then adding *P. lobata* slices and stirring constantly until the surface of *P. lobata* turns golden brown color. Finally, the burnt bran was filtered out, and the filtrate was allowed to cool. Orthogonal experiments were designed based on heating heat (weak medium, medium, high), preheating time (10 s, 15 s, 20 s), stir-fried with bran time (1 min, 2 min, 3 min), and the amount of wheat bran (20, 30, 40%), the design scheme were shown in Tables 1, 2. The detection method for heavy metals was based on the previously mentioned text.

**Table 1:** *Pueraria lobata* stir-fried with bran.

Horizontal	Factor			
	A: Heat fire	B: Preheating time (s)	C: Fried bran time (min)	D: The amount of wheat bran (%)
1	Weak medium	10	1	20
2	Medium	15	2	30
3	High	20	3	40

**Table 2:** Orthogonal design table of *P. lobata.*

Sample	Factor			
	A	B	C	D
1	1	1	1	1
2	1	2	2	2
3	1	3	3	3
4	2	1	2	3
5	2	2	3	1
6	2	3	1	2
7	3	1	3	2
8	3	2	1	3
9	3	3	2	1

### 2.7 Statistical analyses

Raw data were processed using Microsoft Excel (Microsoft Corporation, Redmond, WA, United States). Statistical analyses and charting were performed using OriginPro 2023 software (OriginLab Corporation, Northampton, MA, United States). Pairwise comparisons were analyzed using Student’s *t*-test. Differences were considered statistically significant at *p* < 0.05. All reported results are the averages of three identical experimental runs.

## 3 Results and discussion

### 3.1 Effects of different boiling processes on total polysaccharides content in *Pueraria lobata* soup

**Figure 1:**
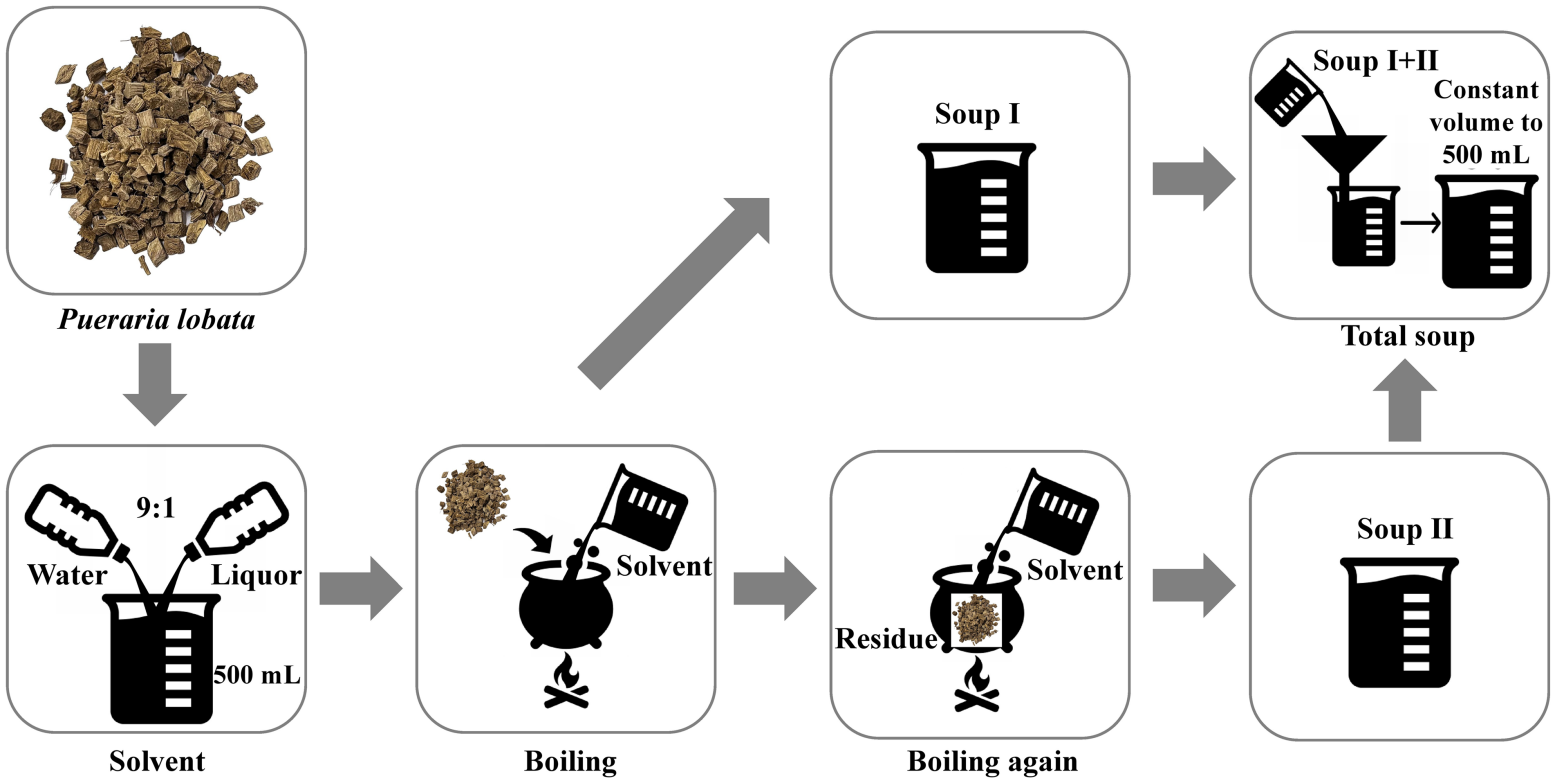
The operation flowchart of boiling processes.

**Figure 2:**
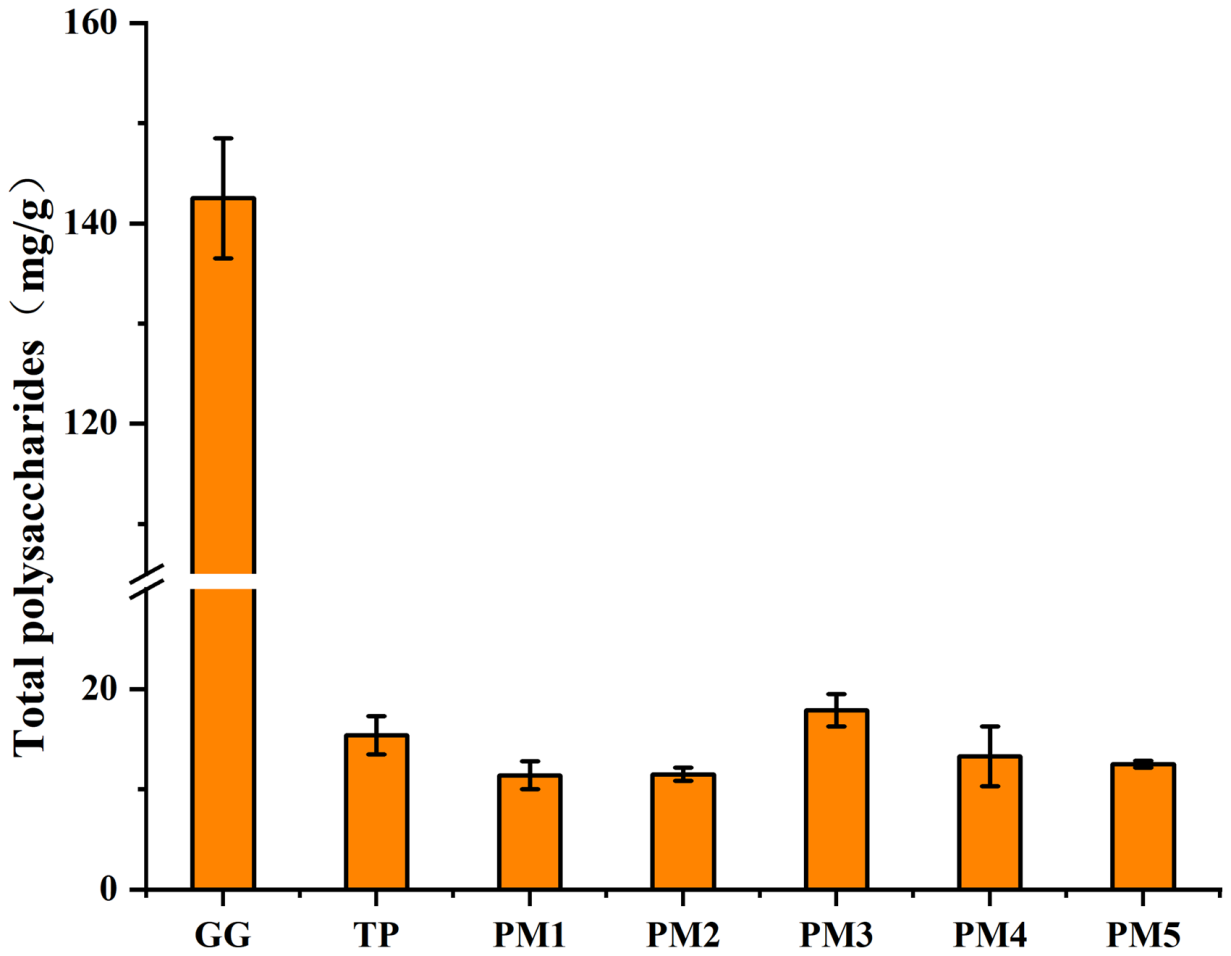
Total polysaccharide content in *P. lobata* and its decoction. GG, raw *P. lobata*; TP, traditional process; PM1, processing method 1; PM2, processing method 2; PM3, processing method 3; PM4, processing method 4; PM5, processing method 5. Significant differences between traditional process and other processes (Student’s *t*-test): *p < 0.05. Data are mean ± standard deviation (*n* = 3, corresponding to three biological replicates).

The flowchart of the boiling process of *P. lobata* slices used in this study is shown in Figure 1. The effect of different boiling processes on total polysaccharide retention was assessed by determining the total polysaccharide content in *P. lobata*. As shown in Figure 2, the polysaccharide content in the decoction prepared by the traditional process in *P. lobata* was 15.4 mg/g, accounting for 10.8% of the total polysaccharide content in *P. lobata* (142.5 mg/g). The highest total polysaccharide content, 17.9 mg/g, was found in the decoction of PM3, while the lowest was 11.4 mg/g in PM1. The total polysaccharide contents for PM2, PM4, and PM5 were 11.5 mg/g, 13.3 mg/g, and 12.5 mg/g, respectively. High temperature can lead to the degradation of polysaccharides, which is consistent with previously reported studies (17, 18). We adopted PM3 to optimise the preparation process of *P. lobata* decoction and obtained *P. lobata* decoction with high polysaccharide content.

Polysaccharides are one of the main active ingredients in traditional Chinese medicine (19). Polysaccharides have different pharmacological effects such as immune modulating, antitumour, antioxidant, and antiviral (20). Previously, Yao et al. showed that the content of polysaccharide components in shiitake mushrooms could be reduced by steaming, boiling, and air-frying for 5–20 min. In addition, the loss of crude polysaccharides and adenosine monophosphate was minimized by roasting for 5–15 min (21). In this study, we analyzed the total polysaccharide content of *P. lobata* and its decoction under different steaming and boiling conditions. The results showed that the total polysaccharide content loss in *P. lobata* was the lowest in processing method 3. One potential explanation for these results is that when water is used as a solvent, polysaccharides dissolve more easily in water than in alcohol or vinegar. This is due to the polarity of water and its ability to form hydrogen bonds, which reduces the loss of total polysaccharide content during the decoction process (22). In addition, under the same solvent conditions, the total polysaccharide content of the decoction steamed using the electric heating pads may be higher than that of the traditional Chinese medicine soup pot method.

### 3.2 Effects of different boiling processes on heavy metal content in *Pueraria lobata* soup

Heavy metal pollution not only affects the quality of traditional Chinese medicinal materials but also degrades the environment and threatens human life and health through the food chain (23, 24). Lead (Pb), Cadmium (Cd), Chromium (Cr), Copper (Cu), and Arsenic (As) are widely recognized as harmful heavy metals to the human body (25). The contamination of Chinese medicinal materials by heavy metals is a serious issue that must be addressed (26). In this study, five heavy metals (As, Pb, Cd, Cr, and Cu) in *P. lobata* were analyzed.

#### 3.2.1 Pb

**Figure 3:**
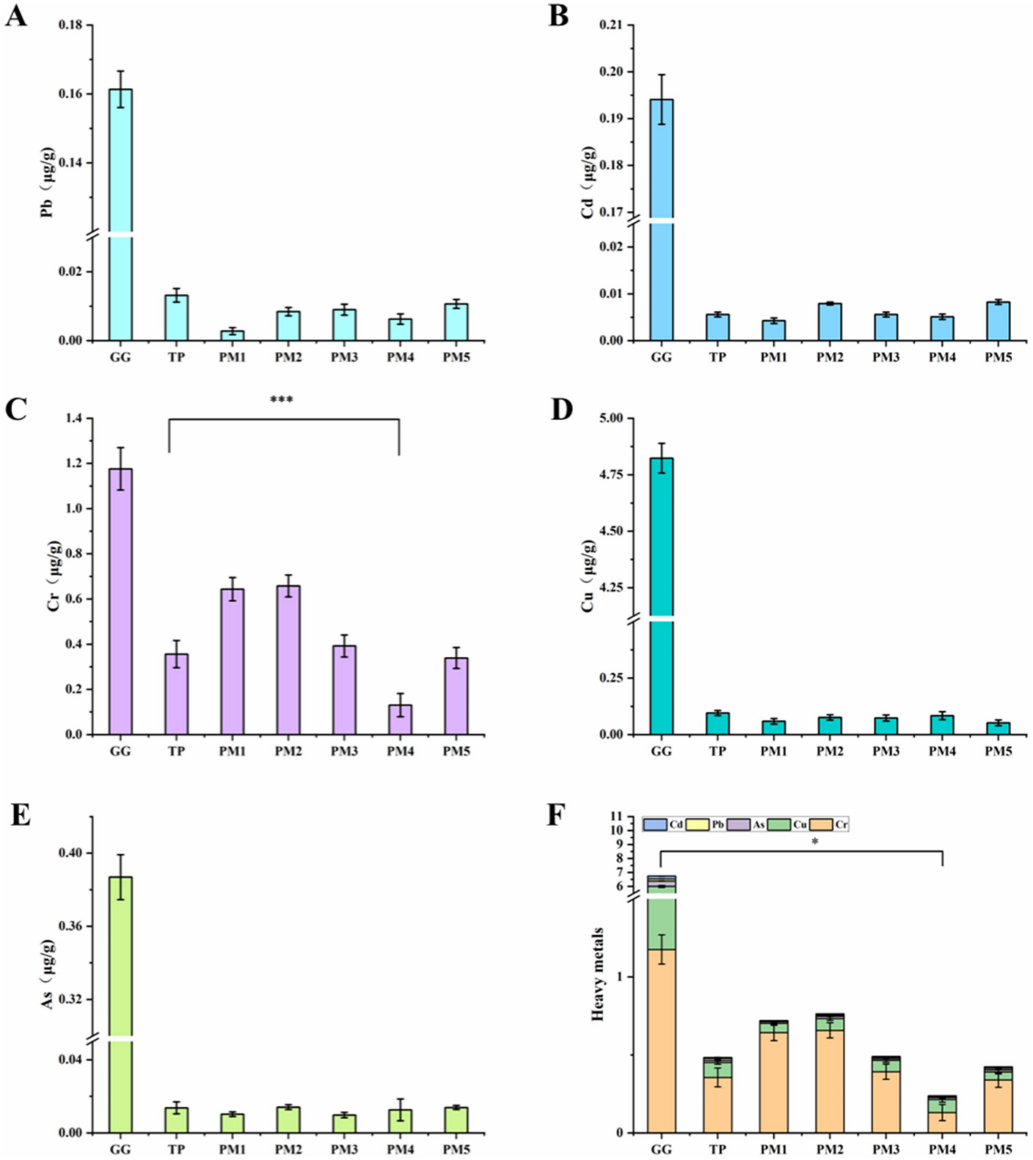
Five heavy metals content in *P. lobata* and its decoction. **(A)** Pb. **(B)** Cd. **(C)** Cr. **(D)** Cu. **(E)** As. **(F)** Total heavy metals content in the soup prepared by optimized process. GG, raw *P. lobata*; TP, traditional process; PM1, processing method 1; PM2, processing method 2; PM3, processing method 3; PM4, processing method 4; PM5, processing method 5. Significant differences between *P. lobata* and other processes (Student’s *t*-test): *p < 0.05, ** *p* < 0.01, ***p < 0.001. Data are mean ± standard deviation (*n* = 3, corresponding to three biological replicates).

Pb, one of the three major heavy metal pollutants, is a harmful heavy metal element that risks human health (27). Pb enters the body mainly through food and drinking water, then causes severe damage to the blood and nervous system (28). The variation of Pb content in *P. lobata* decoctions prepared by different concoctions was shown in Figure 3A. The decoction achieved the best reduction of Pb prepared using PM1 with the Pb content of 2.76 ng/g, which was 79% lower than the Pb content in the traditional process (13.16 ng/g). Moreover, the Pb content in the decoctions of other processing methods was reduced (PM2 with 8.42 ng/g, PM3 with 8.98 ng/g, PM4 with 6.25 ng/g, PM5 with 10.68 ng/g). The results showed that all the optimized processing methods could reduce the Pb content in *P. lobata*. Among them, PM1 was the most effective, and it is speculated that the presence of alcohol is more beneficial in reducing the content of Pb.

#### 3.2.2 Cd

Cd is a highly toxic substance and a recognized carcinogen. In addition, long-term accumulation of Cd also causes a risk to the bones and kidneys (29, 30). Variations in the Cd content of *P. lobata* decoctions prepared using different processing methods were shown in Figure 3B. PM1 was the most effective method for reducing Cd levels in *P. lobata*. The Cd content in the decoction prepared by PM1 was 4.25 ng/g, which was 23% lower than the Cd content (5.58 ng/g) in the decoction prepared by the traditional process. Except for PM1 and PM4 (5.1 ng/g) processing methods, the Cd content in the decoctions prepared by other processing methods was higher than that of the traditional process (PM2 with 7.93 ng/g, PM3 with 5.59 ng/g, PM5 with 8.24 ng/g). There was no direct relationship between solvent addition and Cd content, and the overall Cd content in the electric heating method was slightly lower than that in the decoction pot. Furthermore, the addition of vinegar did not significantly affect the reduction of Cd.

#### 3.2.3 Cr

The excessive intake of Cr can damage the kidneys, liver, and nervous system and cause skin irritation and ulcers (31, 32). Variations in the Cr content of *P. lobata* decoctions prepared using different processing methods were shown in Figure 3C. PM4 showed the best reduction of Cr from *P. lobata* with a residual content of 130.37 ng/g. The Cr content in the PM4 processed decoction was reduced by 79.7% compared to the traditional process (355.84 ng/g). The Cr content in the decoctions prepared by other processing methods was higher than that of the traditional process (PM1 with 643.53 ng/g, PM2 with 657.53 ng/g, PM3 with 392.31 ng/g, and PM5 with 338.85 ng/g). PM4 was the most effective method for reducing Cr, likely due to the superiority of the electric heating method over the frying pan method.

#### 3.2.4 Cu

Cu is an essential component of the human body and has a variety of functions, including promoting blood circulation and metabolism (8). However, the long-term exposure to Cu can lead to toxicity, allergic reactions, and liver damage (33). Variations in the Cu content of *P. lobata* decoctions prepared using different processing methods were shown in Figure 3D. PM5 exhibited the best reduction of Cu. The Cu content in the obtained decoction was 52.1 ng/g, 45.6% lower than that of the traditional process (95.8 ng/g). The other processing methods were also better than the traditional process for Cu reduction (PM1 with 58.91 ng/g, PM2 with 75.63 ng/g, PM3 with 73.47 ng/g, and PM4 with 83.69 ng/g).

#### 3.2.5 As

As is a heavy metal classified by the International Agency for Research on Cancer as a human carcinogen (34). Although trace amounts of As are present in normal human tissues, excessive As can cause damage to blood vessels, eyes, respiratory tract, and skin, potentially leading to death (35). Variations in As content in *P. lobata* decoctions prepared using different processing methods were shown in Figure 3E. PM1 (10.26 ng/g) and PM3 (9.78 ng/g) showed better reduction of As than the traditional process (13.69 ng/g), while the other methods showed comparable reduction of As to the traditional process (PM2 with 14.1 ng/g, PM4 with 12.61 ng/g and PM5 with 13.91 ng/g). The results indicated that different processing methods did not effectively reduce As, and no significant difference was detected in the As content in the decoctions, regardless of the solvent used or the processing method employed.

Based on the analysis of the above results, the content of the five heavy metals in *P. lobata* varied with the processing methods. The Pb, Cd, Cr, Cu, and As contents in the decoction prepared using PM4 and PM5 were significantly lower than those of raw *P. lobata* and the decoction of traditional process. Especially, the content of five heavy metals in PM4 treated *P. lobata* slices was significantly reduced by 96.5% (*p* < 0.05). Compared with the traditional process, the heavy metal content in the PM4 decoction decreased by 50%. This indicates that boiling white wine is beneficial in reducing heavy metals from *P. lobata*, a trend that has also been observed in other common Chinese herbal medicines. Previously, He et al. showed that the total heavy metals (As, Pb, Cd, Cr, and Cu) content of *Lilii Bulbus* soup treated with a water-to-white wine ratio of 9:1 was reduced by 33.5% compared to the decoction of traditional process (12). Similarly, Deng et al. found that flavonoids and fraxinones’ contents significantly increased after the *Dictamni cortex* was concocted with wine (36). The results of this study indicate that the optimal treatment for *P. lobata* is boiling with water and white wine, with the PM4 being the best treatment method. The total heavy metal content was only 0.24 μg/g, significantly lower than the total heavy metal content of *P. lobata* slices (6.74 μg/g).

### 3.3 Effects of different boiling processes on total isoflavone content in *Pueraria lobata* soup

**Figure 4:**
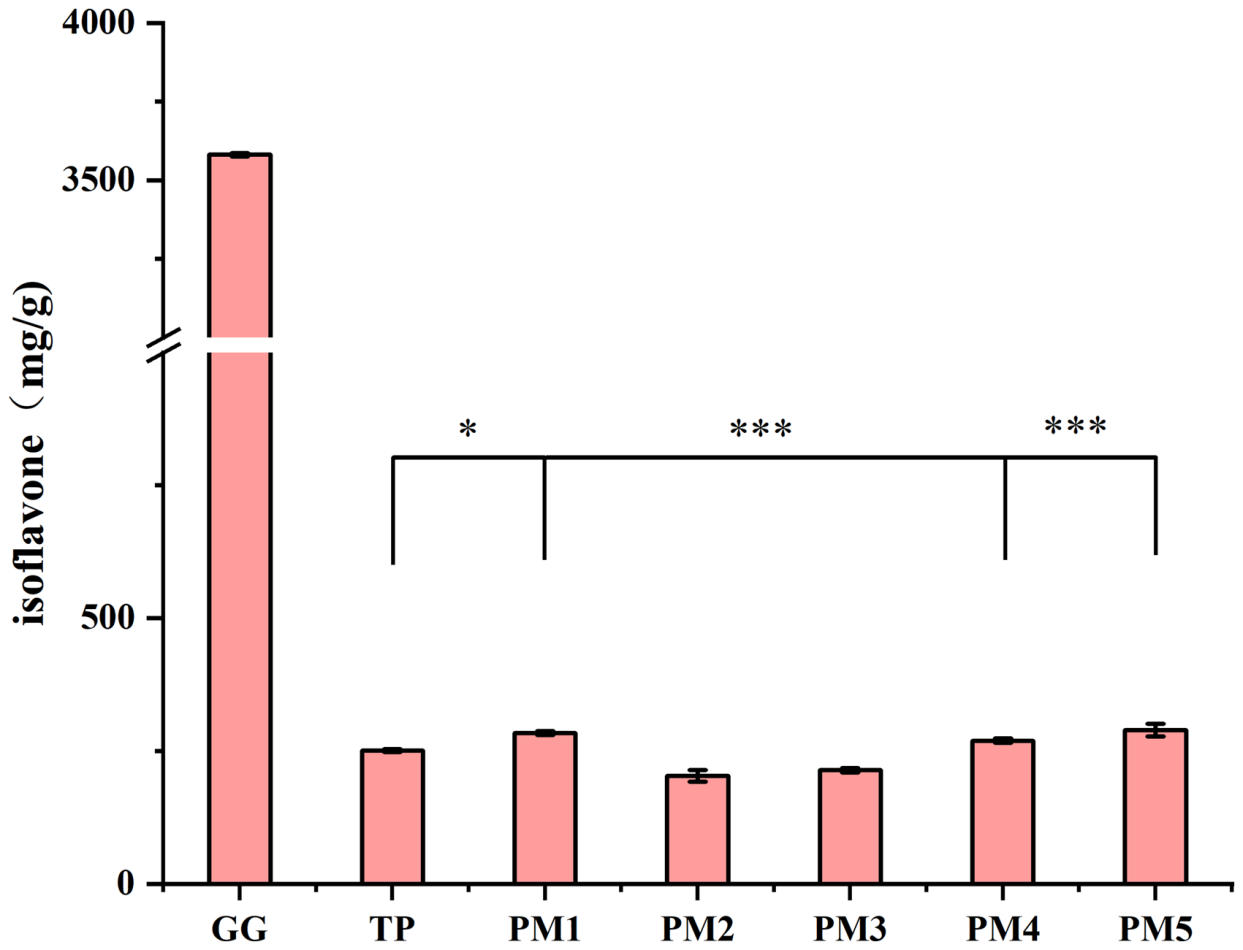
Total isoflavone content in *P. lobata* and its decoction. GG, raw *P. lobata*; TP, traditional process; PM1, processing method 1; PM2, processing method 2; PM3, processing method 3; PM4, processing method 4; PM5, processing method 5. Significant differences between traditional process and other processes (Student’s *t*-test): * *p* < 0.05, ** *p* < 0.01, *** *p* < 0.001. Data are mean ± standard deviation (*n* = 3, corresponding to three biological replicates).

To evaluate the effects of different processing methods on isoflavones, the isoflavone content of *P. lobata* decoctions prepared using different processing methods was determined. As shown in Figure 4, the isoflavone content in *P. lobata* was 3581.5 mg/g. In contrast, the isoflavone content in the decoction prepared by the traditional process was 250.8 mg/g, accounting for 7% of the isoflavone content in *P. lobata*. Among the processing methods, PM1 (283.9 mg/g), PM4 (269.5 mg/g), and PM5 (289.6 mg/g) exhibited higher isoflavone contents compared to that of the traditional process. There was a significant increase of 13.4% in isoflavone content by PM5. In contrast, the other processing methods (PM2 with 203.6 mg/g and PM3 with 213.8 mg/g) exhibited a slight decrease in isoflavone content compared to that of the traditional process, which could be attributed to the high temperature heating that caused the hydrolysis of isoflavones, thus resulting in no significant increase in the isoflavone content (37).

Isoflavones, as the main bioactive compounds in *P. lobata*, have various pharmacological effects (33). Wang et al. found that ambient temperature and moisture content could affect the stability of daidzin in *Astragalus membranaceus* slices (38, 39). In this work, PM5 only retained 7% of the isoflavone content in *P. lobata*, suggesting that heating may lead to the degradation of isoflavones in *P. lobata*.

In this study, although the decoction treated by PM3 had the highest total polysaccharide content, PM3 was not as effective as PM4 in reducing the heavy metal content and retaining the isoflavone content. The optimal processing method, PM4, significantly improved the isoflavone content in *P. lobata* (*p* < 0.001) and reduced the levels of the five heavy metals (*p* < 0.05), resulting in the production of 13.2 mg/g polysaccharides, 0.21 μg/g of the five heavy metals and 269.5 mg/g of isoflavones in the decoction. The total heavy metal content of the decoction of *P. lobata* treated by PM4 was reduced by 96.5%, and its isoflavone content was increased to 7.5% compared to the traditional process. Enhancing the content of isoflavones in the decoction can enhance the pharmacological activity of the decoction and promote the application of *P. lobata* decoction in the food industry (40).

### 3.4 The impact of stir-fried with bran on the heavy metal content in *Pueraria lobata* slices

**Figure 5:**
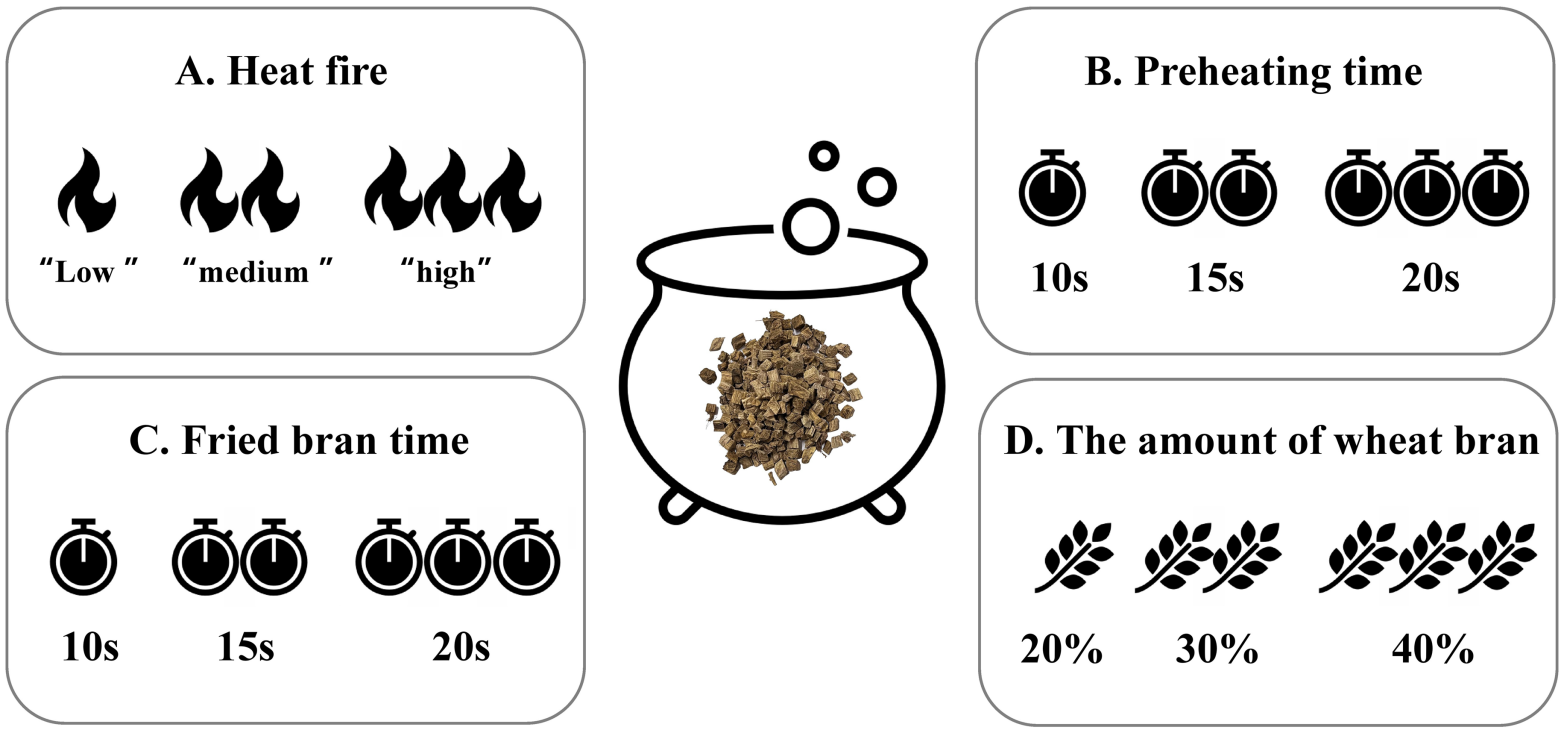
The operation flowchart of stir-fried with bran.

**Figure 6:**
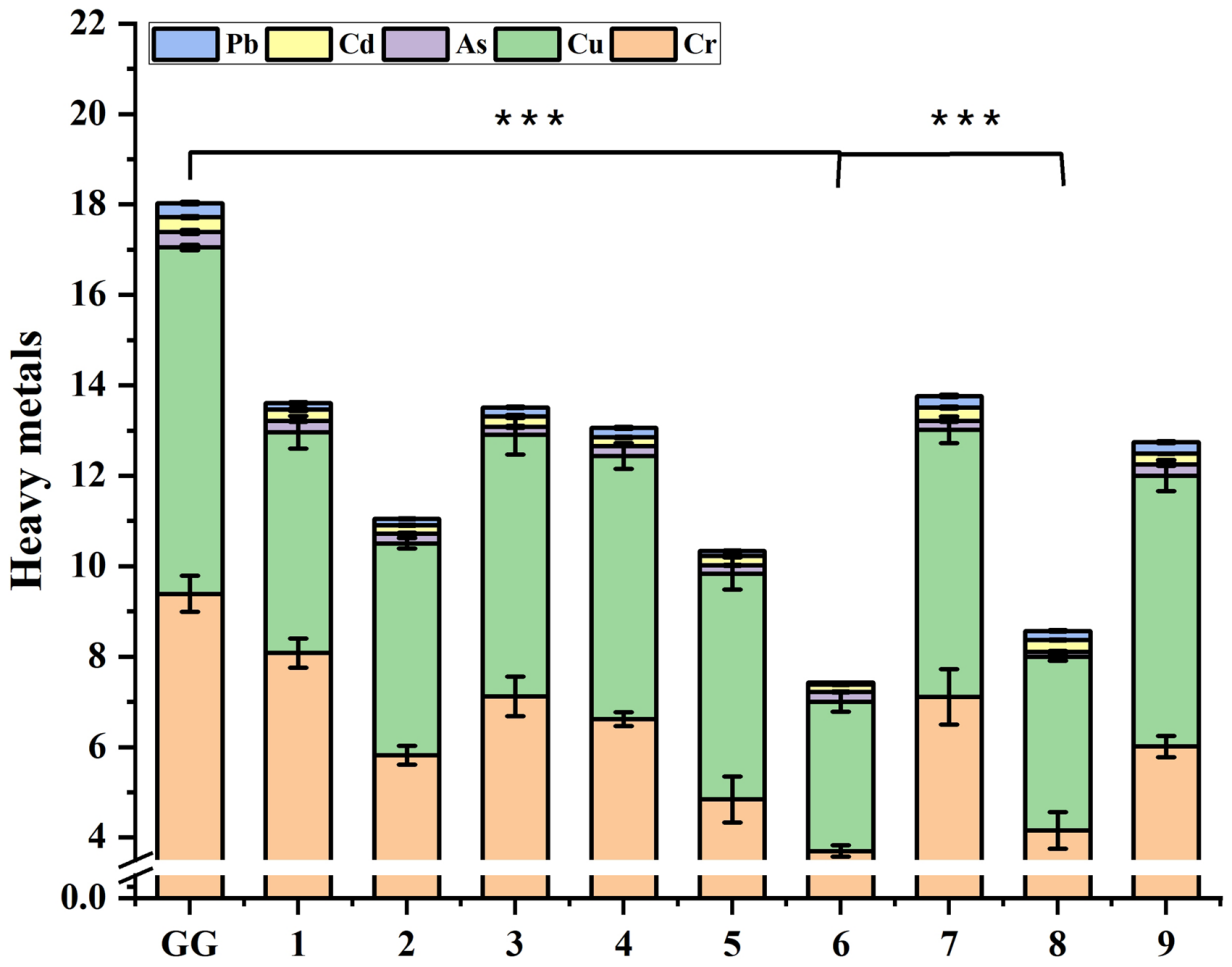
Comparison of the treatment effect of heavy metal concentration in *P. lobata* under different processing processes. Significant differences between the various stir fried with bran processes and the control process (Student’s *t*-test): * *p* < 0.05, ** *p* < 0.01, *** *p* < 0.001. Data are mean ± standard deviation (*n* = 3, corresponding to three biological replicates).

The “Fu Chao” method, a traditional processing technique in Chinese herbal medicine, involves mixing and stir-fried medicinal herbs with wheat bran (41). This process enhances the therapeutic effects of the herbs and tones the spleen (41). In this study, we investigated the effects of different stir-fried with bran processes on the heavy metal content in *P. lobata*. A flowchart of the process for stir-fried with bran of *P. lobata* slices used in this study was shown in Figure 5. As shown in Figure 6, the content of all five heavy metals (Pb, Cd, Cr, Cu, As) in the stir-fried with bran of *P. lobata* was reduced compared with the untreated *P. lobata*. The heavy metal content of *P. lobata* slices processed by methods 6 and 8 was significantly reduced by 58.8 and 52.5%, respectively. In particular, the lowest overall heavy metal content in *P. lobata* was observed in method 6 by heating over medium heat for 20 s and roasting with 30% bran for 1 min.

Stir-fried with bran has many clinical applications in traditional Chinese medicine. For example, Peng et al. determined the optimal processing parameters for stir-fried with bran by conducting an orthogonal variance analysis and principal component analysis on *Fructus Aurantii* (42). The optimal method for stir-fried with bran in this study was method 6, with medium heat, preheating time of 20 s, and 30% bran frying for 1 min. The total heavy metal content of *P. lobata* treated by this method was reduced by 58.8% compared to the control (7.424 μg/g vs. 18.032 μg/g).

## 4 Conclusion

In this study, we optimized the decoction and frying processes of *P. lobata* and evaluated various heating methods and solvents to reduce its heavy metal content. Notably, the continuous heating using a 10:1 ratio of water-to-white wine resulted in a 96.5% reduction in heavy metal levels while increasing the total isoflavone content by 7.5%. Additionally, we refined the stir-frying process using bran through an orthogonal design, which led to a 58.8% reduction in total heavy metals. However, this study has some limitations, including a low retention of polysaccharides and isoflavones. Future research could focus on optimizing the boiling process by adjusting solvent ratios and extraction temperatures. In conclusion, this study will provide the reference for reducing heavy metals in *P. lobata* decoction and *P. lobata* slices, it will enable consumers to obtain safer and more efficient products with a reduced risk of side effects.

## Data Availability

The original contributions presented in the study are included in the article/supplementary material, further inquiries can be directed to the corresponding author.
